# Thyroid Monitoring and Amiodarone-Induced Thyroid Disease in Australian General Practice: A Retrospective Cohort Study

**DOI:** 10.3390/clinpract16030050

**Published:** 2026-02-27

**Authors:** Eva van der Meer, Ven Yin Leong, Gregory M. Peterson, Woldesellassie M. Bezabhe

**Affiliations:** 1 School of Pharmacy and Pharmacology, University of Tasmania, Private Bag 845, Hobart, TAS 7001, Australialeongvy@utas.edu.au (V.Y.L.); woldesellassie.bezabhe@utas.edu.au (W.M.B.); 2 Department of Pharmaceutical Sciences, Division Pharmacoepidemiology & Clinical Pharmacology, Utrecht University, PO Box 80082, 3508 TB Utrecht, The Netherlands

**Keywords:** atrial fibrillation, amiodarone, hypothyroidism, hyperthyroidism, monitoring

## Abstract

**Background:** Australian guidelines recommend conducting thyroid function tests (TFTs) before commencing amiodarone and every six months subsequently. This study sought to investigate thyroid monitoring in Australian general practice patients with atrial fibrillation (AF) who commenced amiodarone. **Methods:** We performed a retrospective observational analysis using a nationwide primary care dataset to examine whether TFTs were conducted according to guidelines following amiodarone initiation in euthyroid patients aged 18 years or older with AF. Secondary outcomes included the prevalence of amiodarone-induced thyroid dysfunction (AITD) and the identification of factors associated with its development. **Results:** In total, 12,932 patients with AF were included. Of these, 1306 (10.1%) had commenced long-term amiodarone. Two hundred twenty-six (17.3%) of the patients commenced on amiodarone did not have any recorded TFT results during an 18-month follow-up period. During follow-up, 18.1% and 4.4% of patients developed hypothyroidism in the amiodarone-treated and amiodarone-untreated groups, respectively (*p* < 0.0001). The corresponding values for hyperthyroidism were 7.3% and 2.5% in the amiodarone-treated and amiodarone-untreated groups, respectively (*p* < 0.0001). In the subset of patients commenced on amiodarone, after controlling for the number of TFTs within the follow-up, the risk factors independently associated with the development of hypothyroidism were baseline thyroid stimulating hormone (TSH) level (adjusted odds ratio/AOR: 3.80 (95% confidence interval: 3.00–4.82)) and the comorbidities heart failure (AOR: 1.64 (1.09–2.46)) and chronic kidney disease (AOR: 2.29 (1.26–4.18)). Baseline TSH (AOR: 0.43 (0.28–0.63)) was significantly associated with the development of hyperthyroidism in patients taking amiodarone. **Conclusions:** AITD was relatively common, occurring in one-quarter of patients within 18 months of initiation of amiodarone. Increased awareness is required amongst both clinicians and patients of the need for regular thyroid monitoring during therapy with amiodarone.

## 1. Introduction

Atrial fibrillation (AF) is the most common cardiac arrhythmia [1,2] and affects over half a million people in Australia [3]. The prevalence of AF is rapidly increasing, especially as the population ages, leading to increased morbidity, mortality, and healthcare costs [4]. Over the last two decades, Australia’s burden of AF-related hospitalisations rose by 295% [5]. It increases the risk of stroke and death five- and two-fold, respectively [6,7], and 20–30% of all stroke cases are due to AF [8].

In addition to stroke prevention, the pharmacological management of AF typically consists of two key strategies: rhythm control and rate control [9,10,11]. Amiodarone, a class III antiarrhythmic drug with all four Vaughan Williams classes of activity, is one of the most effective treatments available for both strategies [9,10,11,12,13,14,15]. It is frequently employed in Australia for AF, especially when other agents are ineffective or contraindicated. However, its use is also problematic due to its long elimination half-life (about 50–60 days) [12,15], tissue accumulation, and the potential for serious adverse effects, particularly involving the thyroid gland, liver, and lungs [10,11,12,13,14,15].

It is believed that 15–20% of patients treated with amiodarone develop amiodarone-induced thyroid dysfunction (AITD) [16], although some studies have reported rates exceeding 30% for hypothyroidism and 20% for hyperthyroidism [16,17,18]. Amiodarone-associated hypothyroidism is more common in areas of sufficient iodine supply, like most of Australia, while hyperthyroidism is more common in areas with iodine deficiency [16]. Explanations for the adverse effects of amiodarone on the thyroid gland include its high iodine content (approximately 37% by weight) and structural similarity to thyroxine, which can produce interference with thyroid hormone production, conversion, and receptor binding [16,19].

To screen for thyroid dysfunction, international and Australian guidelines recommend performing thyroid function tests (TFTs) prior to initiating amiodarone and six-monthly thereafter [11,12,13,14]. While recommendations have been made, compliance with testing guidelines within clinical practice in Australia is unknown. The primary objective of the study was therefore to determine whether TFTs had been conducted in accordance with Australian guidelines in adult patients with AF beginning amiodarone therapy, i.e., three or more tests within 18 months of commencement of amiodarone. We hypothesised that this would not be common practice. Secondary objectives were estimating the prevalence of newly developed thyroid dysfunction (hypothyroidism or hyperthyroidism) and identifying associated risk factors.

## 2. Materials and Methods

This retrospective, observational analysis used primary care data obtained from the National Prescribing Service (NPS) MedicineWise’s dataset (MedicineInsight), collected between 1 January 2009 and 31 December 2018. MedicineInsight includes de-identified patient information, clinical data, prescription medications, and pathology test results from patients visiting participating general practices across all states and territories in Australia. In 2021, MedicineInsight held patient data from 471 general practices covering approximately 9.6% of the population of Australia [20]. Demographic characteristics of the patients are representative of the broader Australian population in terms of age, gender and socioeconomic status [21]. Detailed information about MedicineInsight has been previously published [22]. Following the closure of NPS MedicineWise, MedicineInsight is now under the custodianship of the Australian Commission on Safety and Quality in Health Care [23].

**Figure 1:**
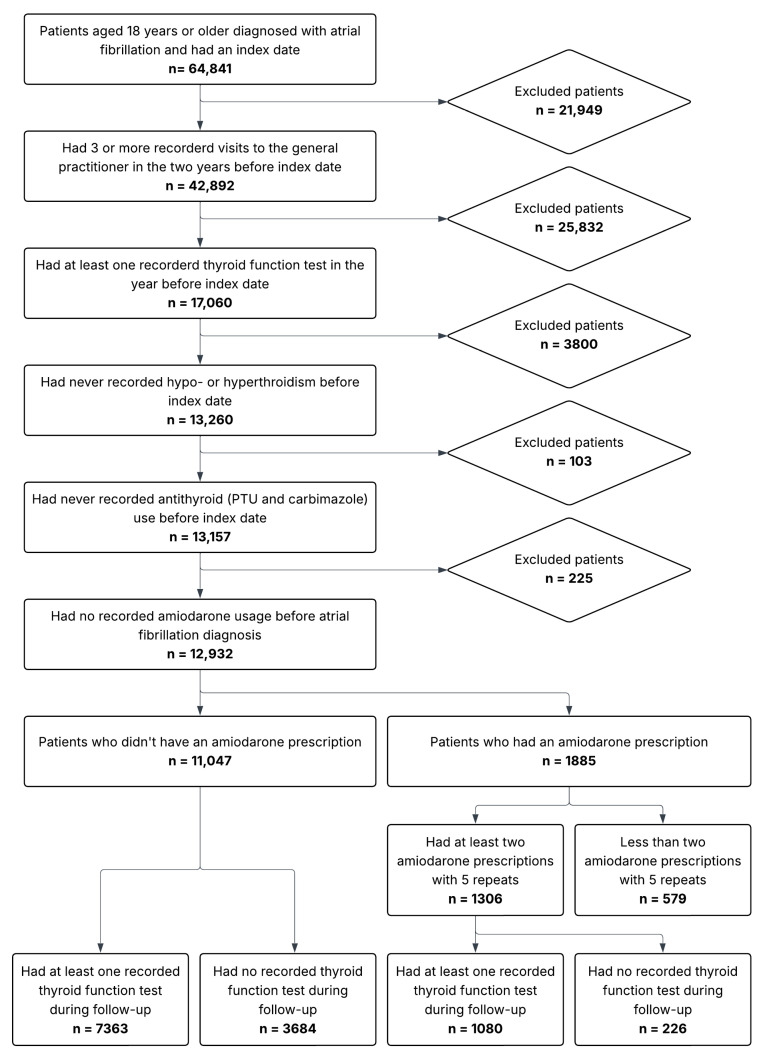
Flowchart of the patient inclusion process.

Patients aged 18 years and older with AF were identified using MedicineInsight condition flags (Figure 1) [24]. The index date was considered that of the initial amiodarone prescription for the amiodarone arm and the date of AF diagnosis for the control arm. Eligibility required at least three general practitioner (GP) consultations over the previous two years, no recorded diagnoses of thyroid disease, and at least one recorded TFT (with normal results) in the year prior to the index date to confirm engagement in care and to exclude a baseline thyroid disorder. Age was assessed at the index date. The following comorbidities were ascertained within 12 months prior to the index date utilising MedicineInsight condition flags: congestive heart failure (CHF), hypertension (HTN), coronary heart disease (CHD), diabetes mellitus, peripheral vascular disease, chronic kidney disease (CKD), venous thromboembolism (VTE), atrial flutter, arthritis, asthma, depression, cancer, chronic obstructive pulmonary disease (COPD), and dementia [24]. The CHA_2_DS_2_-VASc stroke risk score was also calculated for each patient [25].

Patients who had commenced amiodarone were only included if the therapy was likely to be long-term. That is, they must have been prescribed at least two prescriptions with a minimum of five repeats, sufficient for 12 months of therapy.

We grouped the smaller Australian territories with their most closely aligned state (Northern Territory with South Australia and Australian Capital Territory with New South Wales). The Australian Bureau of Statistics (ABS) Index of Relative Socio-Economic Advantage and Disadvantage (IRSAD) is an indicator that summarises information obtained from census data about the socioeconomic conditions (i.e., income, education, employment, occupation and housing variables) of people and households within specified geographic areas, with higher scores indicating more advantaged areas [26]. With the IRSAD as one component, the ABS developed the socio-economic indexes for areas (SEIFA) quintile index, which ranks areas in Australia from 1 (most disadvantaged) to 5 (most advantaged) [26]. The ABS also categorised rurality into five categories using the Accessibility/Remoteness Index of Australia (ARIA) score. These categories include major cities, inner regional, outer regional, remote, and very remote [27].

Hypothyroidism was operationally defined as a thyroid stimulating hormone (TSH) level > 4.0 mU/L with normal or reduced serum thyroxine (T4) (<10 pmol/L), and hyperthyroidism as a TSH < 0.4 mU/L with normal or increased serum T4 (>25 pmol/L) [28]. Subclinical hypothyroidism was not examined.

The distribution of continuous variables was assessed for normality using Kolmogorov–Smirnov tests and Quantile-Quantile plots. Descriptive statistics were used to report demographic and clinical variables, with categorical data expressed as frequencies and percentages, and continuous data as mean values with standard deviations. Independent variables (sex, age, CHA_2_DS_2_-VASc score, baseline TSH, amiodarone maintenance dose, comorbidities, rurality, socioeconomic index, and index year) were assessed by chi-square tests or *t*-tests, as appropriate, to establish unadjusted odds ratios (ORs) for associations with the development of thyroid dysfunction. Values of *p* < 0.05 were included in multivariable logistic regression models to establish adjusted ORs. The number of TFTs performed was included as a covariate in the multiple logistic regression based on our a priori assumption that the frequency of testing would influence the likelihood of being detected with an abnormal TFT result. Statistical analyses were conducted using SAS version 9.4 (SAS Institute Inc., Cary, NC, USA) at the level of statistical significance *p* < 0.05 and with 95% confidence intervals (CIs).

The study was conducted in accordance with the Declaration of Helsinki, and approved by the Human Research Ethics Committee of the University of Tasmania (code: H0017648; approval date: 22 October 2018 and extension as code: 32080; approval date: 19 May 2025). The NPS MedicineWise MedicineInsight Independent Data Governance Committee (28 May 2019) and subsequently the Australian Commission on Safety and Quality in Health Care (13 November 2024) approved the release of data (code: 2018-033). No written consent from the participants was required, given the retrospective use of de-identified health data in a low-risk project (in accordance with Australia’s National Statement on Ethical Conduct in Human Research [29]).

## 3. Results

In total, 12,932 adult patients with AF were included in the study. Of these, 1306 (10.1%) had commenced amiodarone and been prescribed at least two prescriptions with a minimum of five repeats, sufficient for 12 months of therapy. A further 579 patients had been prescribed amiodarone for shorter periods, and these were excluded from further analysis. Two hundred twenty-six (17.3%) of the 1306 patients commenced on long-term amiodarone did not have any recorded TFT results during the 18-month follow-up period (Figure 1). Overall, for the total 1306 patients commencing long-term amiodarone, the mean number of TFTs within 18 months of follow-up was 2.1 ± 1.8.

To determine the prevalence of newly developed thyroid dysfunction, we had to exclude 3910 of the total patients with AF who did not have at least one recorded TFT during the 18-month follow-up period. Of the remaining 8443 patients, 1080 (12.8%) were treated with amiodarone and 7363 (87.2%) were not (Table 1). Females made up 51.9% of all patients, and the cohort had a mean age of 73.8 ± 11.5 years. The baseline characteristics were similar between the groups treated or not treated with amiodarone, although CHF was more common in the amiodarone group (40.4% vs. 25.1%; *p* < 0.0001), probably reflecting the avoidance of beta-blockers in individuals with CHF.

The mean number of TFTs within 18 months of follow-up was 2.5 ± 1.7 for the amiodarone group and 1.7 ± 1.0 for those not prescribed amiodarone (*p* = 0.43). Within 18 months of follow-up, 18.1% and 4.4% of patients had developed hypothyroidism in the amiodarone-treated and amiodarone-untreated groups, respectively (*p* < 0.0001). The corresponding values for hyperthyroidism were 7.3% and 2.5% in the amiodarone-treated and amiodarone-untreated groups, respectively (*p* < 0.0001).

Variables were examined to determine if there were relationships with the development of hypo- or hyperthyroidism. First, the unadjusted and adjusted odds ratios (ORs) were assessed in all the patients with AF, irrespective of long-term treatment with amiodarone, who developed either hypothyroidism (Table S1) or hyperthyroidism (Table S2). Secondly, the unadjusted and adjusted ORs for only the patients who were treated with amiodarone and developed hypothyroidism or hyperthyroidism were calculated (Tables S3 and S4).

After controlling for the number of TFTs within follow-up as a source of ascertainment bias, amiodarone usage (AOR: 4.36 (3.51–5.43)), female sex (AOR: 1.33 (1.07–1.67)), and baseline TSH (AOR: 3.76 (3.33–4.25)) were each independently associated with the development of hypothyroidism in all patients with AF. In the subset of patients commenced on long-term amiodarone, the risk factors which were independently associated with the development of hypothyroidism were baseline TSH (AOR: 3.80 (3.00–4.82)) and the comorbidities CKD (AOR: 2.29 (1.26–4.18)) and CHF (AOR: 1.64 (1.09–2.46)).

After controlling for the number of TFTs within follow-up, amiodarone usage (AOR: 3.27 (2.42–4.38)), female sex (AOR: 1.77 (1.32–2.41)), baseline TSH (AOR: 0.25 (0.19–0.32)), the comorbidity COPD (AOR: 1.44 (1.06–1.94)) and the index years 2012 (AOR: 0.43 (0.20–0.95)) and 2015 (AOR: 0.47 (0.24–0.98)) were each significantly associated with the development of hyperthyroidism in all patients with AF. Baseline TSH (AOR: 0.43 (0.28–0.63)) was significantly associated with the development of hyperthyroidism in those patients taking amiodarone.

## 4. Discussion

In this retrospective, observational study, we evaluated adherence to guidelines on thyroid function monitoring and the prevalence and associated risk factors for thyroid dysfunction in patients treated with amiodarone for AF. International and Australian guidelines recommend performing TFTs prior to initiating amiodarone and six-monthly thereafter [11,12,13,14]. In our study, patients in the amiodarone group received a mean of 2.1 TFTs during the 18-month follow-up, below the recommended three tests. Furthermore, 17.3% of the 1306 patients commenced on long-term amiodarone had no recorded TFTs during follow-up.

After excluding those without any follow-up TFTs, over one-quarter of the patients commenced on amiodarone developed either hypothyroidism (18.1%) or hyperthyroidism (7.3%) within 18 months. These rates, especially for hypothyroidism, are slightly higher than those reported in previous studies [16], although there is considerable variability in the prevalence of AITD between studies [16,17,18]. It should be acknowledged, however, that amiodarone-induced thyrotoxicosis can develop after several years of therapy [16,18], so our 18-month follow-up may have under-estimated its true frequency. The prevalence of thyroid disorders was 3.7 times that in patients not taking amiodarone.

As anticipated, patients who developed thyroid dysfunction had undergone more TFTs than those with normal thyroid function, confirming ascertainment bias (more frequent testing increases the likelihood of detecting abnormal results). While controlling for this effect in the multiple logistic regression, baseline TSH levels were higher in patients who developed hypothyroidism and lower in those who developed hyperthyroidism, suggesting pre-existing subclinical thyroid dysfunction. This pattern was observed both in the overall AF cohort and in the patients treated with amiodarone. It is known that subclinical thyroid abnormalities increase the risk of overt thyroid disease [30,31].

Females were more likely to develop thyroid disorders among all patients, which reflects the well-known association between female sex and autoimmune thyroid diseases [32]. Among other risk factors, COPD was significantly associated with hyperthyroidism in all AF patients. This is consistent with literature indicating that COPD may reduce TSH levels [33,34].

In patients commenced on amiodarone, the comorbidities CKD and CHF were significantly associated with the development of hypothyroidism. Previous studies have also reported higher rates of hypothyroidism in patients with reduced kidney function, potentially due to reduced renal clearance of iodine contained within amiodarone [35,36,37,38], while CHF has been previously associated with amiodarone-induced thyroid dysfunction, perhaps because of more advanced cardiac disease and the associated need for higher dosages of amiodarone in patients with CHF [18,39].

This study has several limitations. To determine the prevalence of newly developed thyroid dysfunction, by necessity, we excluded patients who did not have at least one recorded TFT during the 18-month follow-up period. Given that patients treated with amiodarone had slightly more TFTs performed during follow-up than patients not treated with amiodarone, the aforementioned 3.7-times higher prevalence of newly developed thyroid dysfunction in those who received amiodarone may be an over-estimation of the relative risk due to ascertainment bias. As mentioned, the follow-up period of 18 months may have resulted in an underestimation of the true prevalence of AITD, especially hyperthyroidism. Also, follow-up was discontinued once thyroid dysfunction was diagnosed, which may have missed transitions between hypo- and hyperthyroidism. When examining monitoring of therapy, only regular attendees at the practice and patients who were euthyroid at baseline were included. In addition, it is possible that unmeasured factors, such as a marked change in dietary iodine intake, rather than amiodarone use, may have influenced thyroid function.

It has been previously reported internationally that the safety monitoring of amiodarone is often poor in practice [15], which may be improved through the involvement of pharmacists [40]. In addition, educational interventions directed at both prescribers and patients may be required to highlight the importance of regular ongoing thyroid testing (i.e., six-monthly [11,12,13,14]) during treatment with amiodarone.

## 5. Conclusions

Overall, thyroid function monitoring in Australian primary care patients with AF commencing amiodarone therapy was suboptimal, with approximately one-sixth of the patients having no TFTs performed over an 18-month follow-up period.

## Figures and Tables

**Table 1:** Baseline characteristics of patients with AF who were or were not prescribed amiodarone and who had at least one TFT during follow-up.

Characteristics	Total (*n* = 8443)	No Amiodarone (*n* = 7363)	Amiodarone (*n* = 1080)
**Female sex, *n* (%)**	4382 (51.9)	3854 (52.3)	528 (48.9)
**Age, year, mean ± SD**	73.8 ± 11.5	73.6 ± 11.7	75.1 ± 10.0
**Amiodarone maintenance daily dose in mg, mean ± SD**			181.8 ± 38.6
**Baseline TSH (milli-international units per litre, mU/L), mean ± SD**	1.68 ± 0.80	1.67 ± 0.78	1.78 ± 0.82
**CHA_2_DS_2_-VASc, mean ± SD**	3.5 ± 1.7	3.5 ± 1.8	3.7 ± 1.7
**CHA_2_DS_2_-VASc risk, *n* (%)**			
Mild	576 (6.8)	547 (7.4)	29 (2.7)
Moderate	568 (6.7)	502 (6.8)	66 (6.1)
High	7299 (86.5)	6314 (85.8)	985 (91.2)
**Number of TFTs within 18 months of follow-up, mean ± SD**	1.8 ± 1.2	1.7 ± 1.0	2.5 ± 1.7
**SEIFA, *n* (%)**			
1	1437 (17.0)	1258 (17.1)	179 (16.6)
2	1609 (19.1)	1392 (18.9)	217 (20.1)
3	2144 (25.4)	1858 (25.2)	286 (26.5)
4	1530 (18.1)	1362 (18.5)	168 (15.6)
5	1675 (19.8)	1448 (19.7)	227 (21.0)
**Rurality, *n* (%)**			
Very remote and remote Australia	93 (1.1)	83 (1.1)	10 (0.93)
Outer regional	920 (10.9)	796 (10.8)	124 (11.5)
Inner regional	2420 (28.7)	2097 (28.5)	323 (29.9)
Major cities	4978 (59.0)	4357 (59.2)	621 (57.5)
**State, *n* (%)**			
NSW	3339 (39.5)	2934 (39.8)	405 (37.5)
VIC	1855 (22.0)	1604 (21.8)	251 (23.2)
QLD	1446 (17.1)	1252 (17.0)	194 (18.0)
WA	700 (8.3)	604 (8.2)	96 (8.9)
SA	210 (2.5)	186 (2.5)	24 (2.2)
TAS	664 (7.9)	572 (7.7)	92 (8.5)
ACT	148 (1.8)	136 (1.8)	12 (1.1)
NT	81 (1.0)	75 (1.0)	6 (0.56)
**Index year, *n* (%)**			
2009	275 (3.3)	237 (3.2)	38 (3.5)
2010	440 (5.2)	375 (5.1)	65 (6.0)
2011	636 (7.5)	555 (7.5)	81 (7.5)
2012	808 (9.6)	692 (9.4)	116 (10.7)
2013	981 (11.6)	844 (11.5)	137 (12.7)
2014	1156 (13.7)	1009 (13.7)	147 (13.6)
2015	1239 (14.7)	1080 (14.7)	159 (14.7)
2016	1352 (16.0)	1192 (16.2)	160 (14.8)
2017	1556 (18.4)	1379 (18.7)	177 (16.4)
**Comorbidities, *n* (%)**			
CHF	2281 (27.0)	1845 (25.1)	436 (40.4)
HTN	6014 (71.2)	5223 (70.9)	791 (73.2)
Diabetes mellitus	2090 (24.8)	1815 (24.7)	275 (25.5)
Peripheral vascular disease	459 (5.4)	390 (5.3)	69 (6.4)
CKD (eGFR < 60 mL/1.73 m^2^)	444 (5.3)	363 (4.9)	81 (7.5)
VTE	476 (5.6)	415 (5.6)	61 (5.6)
Atrial flutter	418 (5.0)	317 (4.3)	101 (9.4)
Arthritis	5355 (63.4)	4620 (62.7)	735 (68.1)
Asthma	1773 (21.0)	1518 (20.6)	255 (23.6)
Depression	2455 (29.1)	2126 (28.9)	329 (30.5)
Cancer	3720 (44.1)	3182 (43.2)	538 (49.8)
COPD	1553 (18.4)	1311 (17.8)	242 (22.4)
Dementia	469 (5.6)	428 (5.8)	41 (3.8)

ACT, Australian Capital Territory; CHF, congestive heart failure; CKD, chronic kidney disease; COPD, chronic obstructive pulmonary disease; eGFR, estimated glomerular filtration rate; HTN, hypertension; NSW, New South Wales; NT, Northern Territory; QLD, Queensland; SA, South Australia; SD, standard deviation; SEIFA, socioeconomic indexes for areas; TAS, Tasmania; TFT, thyroid function test; TSH, thyroid stimulating hormone; VIC, Victoria; VTE, venous thromboembolism; WA, Western Australia.

## Data Availability

The data analysed in this study were obtained from NPS MedicineWise MedicineInsight and collected from patients’ records in general practices. Human research ethics committee and NPS MedicineWise MedicineInsight approvals are required to access the data.

## Supplementary Materials

The following supporting information can be downloaded at: https://www.mdpi.com/article/10.3390/clinpract16030050/s1, Table S1: Unadjusted and adjusted ORs of risk factors associated with hypothyroidism in all patients with AF; Table S2: Unadjusted and adjusted ORs of risk factors associated with hyperthyroidism in all patients with AF; Table S3: Unadjusted and adjusted AORs of risk factors associated with hypothyroidism in patients taking amiodarone; Table S4: Unadjusted and adjusted AORs of risk factors associated with hyperthyroidism in patients taking amiodarone.
