# Supplementary material for: Thyroid Monitoring and Amiodarone-Induced Thyroid Disease in Australian General Practice: A Retrospective Cohort Study
Source: Clin Pract. 2026 Feb 27;16(3):50. doi: 10.3390/clinpract16030050 (PMC13025236; doi:10.3390/clinpract16030050)
Supplement: Supplementary file 1 [file clinpract-16-00050-s001.zip › clinpract-4047556-supplementary.pdf]

Supplementary Tables

Table S1. Unadjusted and adjusted ORs of risk factors associated with hypothyroidism in all patients with AF.

Characteristics	Unadjusted OR (95% CI)	p-value	Adjusted OR (95% CI)	p-value
**Female sex**	1.40 (1.17-1.68)	<0.001	1.33 (1.07-1.67)	0.012
**Age in years at baseline**	1.02 (1.01-1.02)	<0.001	1.00 (0.99-1.02)	1
**Baseline TSH (milli-international units per litre, mU/L)**	3.58 (3.20-4.00)	<0.001	3.76 (3.33-4.25)	<0.001
**CHA_2DS_2-VASc score**	1.14 (1.09-1.20)	<0.001	1.01 (0.92-1.10)	0.827
**Number of TFTs within 18 months of follow-up**	4.36 (3.53-5.39)	<0.001	3.91 (3.11-4.92)	<0.001
**Amiodarone prescribed**	5.11 (4.22-6.19)	<0.001	4.36 (3.51-5.43)	<0.001
**SEIFA**				
1	0.87 (0.65-1.17)	0.353	1.05 (0.80-1.38)	0.726
2	0.91 (0.68-1.21)	0.521	1.10 (0.83-1.46)	0.508
3	1.05 (0.81-1.36)	0.712	1.26 (0.89-1.78)	0.191
4	0.91 (0.68-1.21)	0.521	1.14 (0.64-2.00)	0.652
5	Ref			
**Rurality**				
Very remote, remote, and outer regional	0.94 (0.70-1.27)	0.683		
Inner regional	0.95 (0.72-1.25)	0.715		
Major cities	Ref			
**State**				
NSW and ACT	Ref		Ref	
VIC	0.98 (0.77-1.26)	0.872	1.05 (0.80-1.38)	0.726
QLD	1.20 (0.94-1.55)	0.153	1.10 (0.83-1.46)	0.508
WA	1.52 (1.12-2.07)	0.008	1.26 (0.89-1.78)	0.191
SA and NT	1.10 (0.66-1.83)	0.714	1.14 (0.64-2.00)	0.652
TAS	1.57 (1.15-2.14)	0.004	1.33 (0.94-1.90)	0.112
**Index year**				
2009	Ref		Ref	
2010	1.14 (0.58-2.24)	0.704		
2011	1.38 (0.74-2.56)	0.309		
2012	1.52 (0.83-2.75)	0.171		
2013	1.36 (0.75-2.46)	0.310		
2014	1.23 (0.68-2.22)	0.493		
2015	1.26 (0.70-2.25)	0.438		
2016	0.91 (0.50-1.65)	0.757		
2017	1.15 (0.65-2.06)	0.635		
**Comorbidities**				
CHF	1.73 (1.44-2.08)	<0.001	1.27 (1.00-1.61)	0.049
HTN	1.03 (0.85-1.26)	0.768		
CHD	1.24 (1.02-1.49)	0.026	1.04 (0.84-1.30)	0.724
Diabetes mellitus	1.20 (0.98-1.46)	0.073		
Peripheral vascular disease	1.30 (0.91-1.85)	0.147		

CKD (eGFR<60 mL/1.73 m^2)	1.73 (1.25-2.40)	<0.001	1.17 (0.80-1.71)	0.4178
VTE	1.01 (0.69-1.48)	0.959		
Atrial flutter	1.21 (0.83-1.77)	0.324		
Arthritis	1.20 (1.00-1.45)	0.054		
Asthma	1.03 (0.83-1.28)	0.7891		
Depression	1.22 (1.01-1.47)	0.037	1.10 (0.88-1.36)	0.391
Cancer	1.14 (0.95-1.36)	0.152		
COPD	1.34 (1.09-1.66)	0.006	1.20 (0.94-1.53)	0.142
Dementia	0.85 (0.56-1.29)	0.445		

Table S2. Unadjusted and adjusted ORs of risk factors associated with hyperthyroidism in all patients with AF.

**Characteristics**	**Unadjusted OR (95% CI)**	**p-value**	**Adjusted OR (95% CI)**	**p-value**
**Female sex**	1.95 (1.50-2.54)	<0.001	1.77 (1.32-2.41)	<0.001
**Age in years at baseline**	1.01 (1.00-1.02)	0.049	1.00 (0.99-1.02)	1
**Baseline TSH (milli-international units per litre, mU/L)**	0.25 (0.20-0.32)	<0.001	0.25 (0.19-0.32)	<0.001
**CHA_2DS_2-VASc score**	1.15 (1.07-1.23)	<0.001	1.01 (0.90-1.14)	0.869
**Number of TFTs within 18 months of follow-up**	7.94 (5.54-11.4)	<0.001	6.94 (4.86-10.21)	<0.001
**Amiodarone prescribed**	3.72 (2.83-4.89)	<0.001	3.27 (2.42-4.38)	<0.001
**SEIFA**				
1	1.39 (0.94-2.07)	0.102		
2	1.29 (0.87-1.90)	0.201		
3	0.93 (0.63-1.38)	0.717		
4	0.95 (0.62-1.45)	0.813		
5	Ref			
**Rurality**				
Very remote, remote, and outer regional	1.41 (0.98-2.02)	0.063		
Inner regional	1.22 (0.93-1.62)	0.160		
Major cities	Ref			
**State**				
NSW and ACT	Ref		Ref	
VIC	1.18 (0.87-1.59)	0.282		
QLD	0.57 (0.37-0.87)	0.009		
WA	0.71 (0.42-1.21)	0.205		
SA and NT	1.06 (0.55-2.04)	0.950		
TAS	1.05 (0.66-1.67)	0.837		
**Index year**				
2009	Ref		Ref	
2010	1.18 (0.60-2.30)	0.629	1.20 (0.59-2.57)	0.627
2011	0.68 (0.34-1.35)	0.273	0.73 (0.35-1.57)	0.411
2012	0.44 (0.21-0.89)	0.026	0.43 (0.20-0.95)	0.034
2013	0.52 (0.27-1.01)	0.052	0.59 (0.29-1.24)	0.155
2014	0.52 (0.27-0.99)	0.049	0.55 (0.28-1.14)	0.095
2015	0.47 (0.25-0.90)	0.021	0.47 (0.24-0.98)	0.035
2016	0.67 (0.36-1.23)	0.201	0.72 (0.38-1.45)	0.336
2017	0.57 (0.31-1.06)	0.073	0.62 (0.33-1.26)	0.162
**Comorbidities**				
CHF	1.30 (1.00-1.70)	0.053	0.96 (0.69-1.32)	0.805
HTN	1.10 (0.83-1.46)	0.508		

CHD	1.06 (0.81-1.39)	0.672		
Diabetes mellitus	1.21 (0.92-1.59)	0.172		
Peripheral vascular disease	0.70 (0.37-1.33)	0.274		
CKD (eGFR<60 mL/1.73 m^2)	1.16 (0.68-1.97)	0.584		
VTE	0.95 (0.55-1.65)	0.855		
Atrial flutter	0.61 (0.30-1.24)	0.172		
Arthritis	1.30 (1.00-1.70)	0.053	1.02 (0.77-1.37)	0.893
Asthma	1.01 (0.75-1.37)	0.948		
Depression	1.46 (1.13-1.89)	0.004	1.20 (0.91 -1.58)	0.195
Cancer	1.00 (0.78-1.28)	1		
COPD	1.63 (1.23-2.16)	<0.001	1.44 (1.06-1.94)	0.018
Dementia	1.11 (0.66-1.86)	0.693		

Table S3. Unadjusted and adjusted AORs of risk factors associated with hypothyroidism in patients taking amiodarone.

**Characteristics**	**Unadjusted OR (95% CI)**	**p-value**	**Adjusted OR (95% CI)**	**p-value**
**Female sex**	1.41 (1.03-1.93)	0.032	1.25 (0.84-1.87)	0.274
**Age in years at baseline**	1.01 (0.99-1.03)	0.325		
**Amiodarone daily maintenance dose (mg)**	1.00 (1.00-1.01)	1	1.00 (0.99-1.01)	1
**Baseline TSH (milli-international units per litre, mU/L)**	3.64 (2.92-4.54)	<0.001	3.80 (3.00-4.82)	<0.001
**CHA_2DS_2-VASc score**	1.15 (1.05-1.26)	0.003	1.05 (0.92-1.20)	0.472
**Number of TFTs within 18 months of follow-up**	2.79 (1.90-4.08)	<0.001	3.03 (1.98-4.65)	<0.001
**SEIFA**				
1	1.01 (0.60-1.69)	0.970		
2	1.02 (0.62-1.66)	0.937		
3	1.15 (0.74-1.80)	0.538		
4	0.74 (0.43-1.29)	0.283		
5	Ref			
**Rurality**				
Very remote, remote, and outer regional	0.97 (0.59-1.60)	0.904		
Inner regional	1.00 (0.71-1.43)	1		
Major cities	Ref			
**State**				
NSW and ACT	Ref		Ref	
VIC	1.05 (0.69-1.62)	0.823	1.10 (0.68-1.77)	0.696
QLD	1.15 (0.73-1.80)	0.544	1.00 (0.60-1.68)	1
WA	1.61 (0.93-2.76)	0.086	1.46 (0.78-2.73)	0.236
SA and NT	1.19 (0.47-3.02)	0.714	0.87 (0.31-2.44)	0.791
TAS	1.79 (1.04-3.09)	0.036	1.39 (0.73-2.66)	0.318
**Index year**				
2009	Ref			
2010	0.92 (0.28-3.08)	0.892		
2011	1.93 (0.65-5.71)	0.236		
2012	1.57 (0.55-4.49)	0.400		
2013	1.62 (0.57-4.57)	0.363		
2014	1.63 (0.58-4.56)	0.353		
2015	1.49 (0.53-4.16)	0.448		

2016	1.65 (0.59-4.59)	0.339		
2017	1.09 (0.39-3.07)	0.870		
**Comorbidities**				
CHF	1.61 (1.18-2.21)	0.003	1.64 (1.09-2.46)	0.017
HTN	0.94 (0.67-1.34)	0.726		
CHD	1.11 (0.81-1.52)	0.516		
Diabetes mellitus	1.29 (0.91-1.82)	0.150		
Peripheral vascular disease	1.23 (0.67-2.23)	0.500		
CKD (eGFR<60 mL/1.73 m^2)	2.31 (1.40-3.82)	0.001	2.29 (1.26-4.18)	0.007
VTE	1.13 (0.58-2.18)	0.717		
Atrial flutter	0.67 (0.37-1.21)	0.185		
Arthritis	1.30 (0.92-1.84)	0.138		
Asthma	1.15 (0.80-1.64)	0.445		
Depression	1.08 (0.77-1.51)	0.654		
Cancer	1.22 (0.89-1.66)	0.211		
COPD	1.15 (0.80-1.66)	0.453		
Dementia	0.50 (0.18-1.44)	0.191		

Table S4. Unadjusted and adjusted AORs of risk factors associated with hyperthyroidism in patients taking amiodarone.

**Characteristics**	**Unadjusted OR (95% CI)**	**p-value**	**Adjusted OR (95% CI)**	**p-value**
**Female sex**	1.15 (0.73-1.83)	0.551	1.02 (0.60-1.74)	0.942
**Age in years at baseline**	1.02 (0.99-1.05)	0.187	1.04 (1.00-1.07)	0.023
**Amiodarone daily maintenance dose (mg)**	1.00 (1.00-1.01)	1	1.00 (0.99-1.01)	1
**Baseline TSH (milli-international units per litre, mU/L)**	0.44 (0.30-0.65)	<0.001	0.43 (0.28-0.63)	<0.001
**CHA2DS2-VASc score**	1.04 (0.91-1.19)	0.567	0.91 (0.75-1.10)	0.334
**Number of TFTs within 18 months of follow-up**	5.95 (2.85-12.52)	<0.001	6.12 (3.05-14.04)	<0.001
**SEIFA**				
1	1.22 (0.61-2.46)	0.576		
2	0.93 (0.46-1.88)	0.840		
3	0.76 (0.38-1.511)	0.436		
4	0.77 (0.35-1.69)	0.515		
5	Ref			
**Rurality**				
Very remote, remote, and outer regional	1.88 (0.98-3.63)	0.059		
Inner regional	1.60 (0.96-2.68)	0.073		
Major cities	Ref			
**State**				
NSW and ACT	Ref			
VIC	1.34 (0.77-2.33)	0.300		
QLD	0.60 (0.28-1.29)	0.190		
WA	0.73 (0.28-1.94)	0.524		
SA and NT	It is not possible to calculate			
TAS	1.30 (0.57-2.96)	0.532		
**Index year**				

2009	Ref			
2010	0.96 (0.21-4.31)	0.958		
2011	1.25 (0.30-5.19)	0.759		
2012	0.57 (0.13-2.52)	0.457		
2013	1.25 (0.30-5.19)	0.759		
2014	1.03 (0.27-3.92)	0.965		
2015	0.66 (0.17-2.64)	0.553		
2016	1.42 (0.39-5.18)	0.595		
2017	1.01 (0.27-3.75)	0.988		
**Comorbidities**				
CHF	0.85 (0.52-1.38)	0.514		
HTN	0.93 (0.56-1.56)	0.781		
CHD	0.85 (0.52-1.38)	0.514		
Diabetes mellitus	0.9 7(0.57-1.67)	0.893		
Peripheral vascular disease	0.58 (0.18-1.92)	0.367		
CKD (eGFR<60 mL/1.73 m2)	1.02 (0.40-2.64)	0.967		
VTE	1.17 (0.45-3.04)	0.747		
Atrial flutter	0.46 (0.17-1.30)	0.135		
Arthritis	0.79 (0.49-1.27)	0.332		
Asthma	0.76 (0.43-1.37)	0.353		
Depression	1.08 (0.66-1.77)	0.760		
Cancer	0.86 (0.54-1.38)	0.529		
COPD	0.97 (0.56-1.71)	0.915		
Dementia	1.63 (0.62-4.32)	0.324		
